# Sildenafil Induced Acute Interstitial Nephritis

**DOI:** 10.1155/2015/731284

**Published:** 2015-09-27

**Authors:** Ryan Burkhart, Nina Shah, Matthew Lewin

**Affiliations:** ^1^William Beaumont Army Medical Center, 5005 N. Piedras Street, El Paso, TX 79920, USA; ^2^ProPath Services, LLP, 1355 River Bend Drive, Dallas, TX 75247, USA

## Abstract

Acute interstitial nephritis (AIN) is characterized by inflammation of the renal interstitium and usually occurs in a temporal relationship with the medication. We present a case of an Asian male who had nephrotic range proteinuria and presented with acute kidney injury. The patient reported an acute change in physical appearance and symptomatology after the ingestion of a single dose of sildenafil. Renal biopsy was notable for minimal change disease (MCD) with acute and chronic interstitial nephritis. Renal replacement and glucocorticoid therapy were initiated. Renal recovery within six weeks permitted discontinuation of dialysis. AIN superimposed on MCD is a known association of NSAID induced nephropathy. The temporal association and the absence of any new drugs suggest that the AIN was most likely due to the sildenafil. NSAIDs are less likely to have caused the AIN given their remote use. The ease of steroid responsiveness would also suggest another cause as NSAID induced AIN is often steroid resistant. The MCD was most likely idiopathic given the lack of temporal association with a secondary cause. As the number of sildenafil prescriptions increases, more cases of AIN may be identified and physician awareness for this potential drug disease association is necessary.

## 1. Introduction

Acute interstitial nephritis (AIN) is a known cause of intrinsic acute kidney injury and is characterized by inflammation of the renal interstitium. AIN has been reported to occur in approximately 1–3% of all renal biopsies, and up to 15–27% when biopsy indication is due to renal failure [1]. Drug induced AIN notably occurs in approximately 70% of cases, with the majority of cases being due to antibiotics, proton pump inhibitors, or NSAIDs [2]. Drug induced AIN usually occurs in a temporal relationship with a medication. While its diagnosis may be apparent based on presentation and ruling out more common causes of acute kidney injury, definitive diagnosis is made by renal biopsy. Given that almost any drug can potentially cause AIN and frequent coinciding polypharmacy, the offending agent can often be difficult to identify.

To the best of our knowledge, there have been no published reports of AIN due to sildenafil. We present a case of biopsy proven AIN likely attributable to sildenafil in an individual who also had minimal change disease (MCD).

## 2. Case Report

This is an 81-year-old Asian male with a known past medical history of erectile dysfunction, chronic kidney disease stage 3a, hypertension, hyperlipidemia, coronary artery disease, gout with chronic allopurinol use for years, and osteoarthritis with remote NSAID use. The patient was admitted with generalized edema, rapid weight gain of 9.1 kg over the previous month, hyperkalemia 5.9 mmol/L (5.9 mEq/L), BUN 17.14 mmol/L (48 mg/dL), and serum creatinine of 327.08 *μ*mol/L (3.7 mg/dL). His baseline serum creatinine was 123.76 *μ*mol/L (1.4 mg/dL) and eGFR was 47 mL/min/1.73 m^2^ by CKD-EPI. He was noted to have 1661.1 mg/mmol proteinuria (14.7 mg/mg), serum BUN 13.57 mmol/L (38 mg/dL), and serum creatinine of 167.96 *μ*mol/L (1.9 mg/dL) two weeks before. The patient specifically noted an acute increase in peripheral and facial edema after ingesting a single dose of sildenafil four days prior to his admission.

His admission medications included lisinopril, diltiazem, atorvastatin, aspirin, allopurinol, tramadol, docusate, and sildenafil. The patient specifically denied any recent NSAID usage or over-the-counter medications. He was previously on sulindac as needed with the last dose thirteen months prior to his presentation. Sildenafil was the only new medication.

On admission, the patient had unremarkable cardiac and pulmonary exams and diffuse bilateral lower extremity edema. Blood pressure was 144/70 mmHg. Chest X-ray noted small bilateral pleural effusions. Renal ultrasound revealed normal parenchyma bilaterally, without evidence of hydronephrosis. Cardiac echo revealed an ejection fraction of 68% with structurally normal valves and chambers.

Additional labs on admission noted a WBC of 5.4 × 10^9^/L with 5.6% eosinophils (normal, 0–7%). Albumin was 26 g/L (2.6 g/dL). Urinalysis was notable for specific gravity of 1.020, blood 4+, and protein 4+. Urine sediment noted 0–2 granular casts/lpf, no cellular casts/lpf, 0–5 nondysmorphic RBCs/hpf, and 0-1 WBC/hpf on microscopy.

Renal biopsy was performed. Twenty-three glomeruli were obtained. Six out of twenty-three glomeruli were obsolescent with capillary tuft collapse and collagen accumulation within Bowmen's space consistent with hypertensive nephrosclerosis. The viable glomeruli were without significant evidence of increased mesangial matrix or mesangial cellularity. There was hyperplasia of the visceral epithelial cells and occasional protein reabsorption granules noted on the PAS stain. The glomerular basement membranes were slightly thickened but without discrete subepithelial spikes, pinholes, deposits, or double contours noted. No definitive segmental sclerotic lesions were identified.

The tubulointerstitium had patchy moderate interstitial inflammation with numerous eosinophils observed consistent with acute interstitial nephritis. There was mild interstitial fibrosis consistent with chronic interstitial nephritis.

Immunofluorescence showed segmental protein reabsorption granular staining for IgM (2+), C3 (2+), albumin (2+), kappa light chain (2+), and lambda light chain (2+). There was nonspecific linear tubal basement membrane staining for albumin (1+). Staining for IgA, IgG, C1q, or fibrinogen was negative. Electron microscopy showed normal cell elements and mesangial matrix. The glomerular basement membranes were normal without subendothelial or subepithelial densities. There was diffuse global foot process effacement with associated microvillus hypertrophy consistent with minimal change disease.

Dialysis was initiated in the setting of progressive decline in renal function. The patient was treated with 1 gram of methylprednisolone for 3 days followed by a prednisone taper over 18 weeks. Renal recovery within six weeks permitted discontinuation of dialysis. Proteinuria decreased to 57.9 mg/mmol (0.512 mg/mg), and serum creatinine returned to its prior baseline of 123.76 *μ*mol/L (1.4 mg/dL).

## 3. Discussion

Initial differential diagnosis included membranous nephropathy, minimal change disease, IgA nephropathy, focal segmental glomerulosclerosis, fibrillary glomerulonephritis, immunotactoid glomerulopathy, amyloidosis, light chain deposition disease, and myeloma kidney. AIN was also on the differential diagnosis for his AKI; however, it would not account for the nephrotic range proteinuria. The renal biopsy was performed in the setting of nephrotic syndrome and AKI of unclear etiology.

Our patient had a very complicated presentation of acute kidney injury and nephrotic syndrome in the setting of multiple medications, two of which have a very well-known association with AIN. His renal biopsy noted MCD, with acute and early chronic interstitial nephritis, which would notably be characteristic of an NSAID induced etiology [3, 4]. We have not been able to find any reports of sildenafil induced AIN. There are multiple factors that would argue against NSAID, or allopurinol induced AIN, and would argue for sildenafil as the culprit agent.

There was a temporal association with sildenafil ingestion and the patient's symptom onset. First exposure to a medication may take weeks for the development of AIN. Medication reexposure may allow for AIN to develop more quickly and usually occurs in 3–5 days [5] but may occur as early as one day [6]. The majority of NSAID induced AIN have been on the medication for approximately 6 months [7]. Our patient's symptoms developed rapidly following the ingestion of sildenafil. AKI was noted 4 days later. Review of his records noted that he was prescribed sildenafil for the first time 15 months before. We suspect that this prior exposure likely primed his immune system to allow for the rapidity of the AIN to develop.

The patient adamantly denied recent consumption of NSAIDs (aside from aspirin) including both over-the-counter or prescription based medications. The patient had previously been on sulindac 150 mg twice a day for his osteoarthritis, with his last consumption thirteen months prior to his presentation. He had been on sulindac for more than two years prior to its cessation. The patient had been on allopurinol for decades and was without evidence of AIN despite continuous allopurinol use. He was also without the characteristic rash and abnormal liver associated tests that often accompany allopurinol induced AIN. The patient was on aspirin 325 mg daily as well which has been described in the literature to be associated with AIN [7]. However, there was no temporal association with the initiation of these medications. The patient remained on aspirin during and after treatment. Despite remaining on aspirin, his renal function returned to baseline and the degree of proteinuria significantly improved. The lack of temporal association here strongly goes against an NSAID or allopurinol induced etiology.

NSAID induced AIN is classically known to be less responsive to corticosteroids [7, 8] and portends a worse prognosis [9]. The patient had MCD as well as AIN and had an excellent clinical response that allowed him to become dialysis independent at 6 weeks. In this case, the corticosteroids were treating his MCD as well as the AIN. His renal function continued to improve back to his baseline. The ease of steroid responsiveness would suggest another etiology as NSAID induced AIN is often steroid resistant.

The patient had evidence of acute and “early” chronic interstitial nephritis on the renal biopsy which refers to the degree of fibrosis. The chronic portion could potentially be attributed to his chronic hypertension, prior NSAID use, or the allopurinol. This may also be secondary to the sildenafil. The renal biopsy was performed approximately 10 days after the ingestion of sildenafil, and 9 days after the onset of the patient's symptoms. Fibrosis has been shown to develop in as little as a week with AIN [5, 10] as part of an inflammatory continuum resulting in fibrogenesis [5]. The interstitial nephritis may have been undergoing its natural progression following the sildenafil exposure, resulting in those chronic changes.

MCD in the elderly is not uncommon and has been reported in roughly 10–15% of cases of nephrotic syndrome in adults [11] and with increased incidence among Asians [12]. While NSAIDS are a common cause of secondary MCD, the most common etiology of MCD is idiopathic. The patient was noted to have nephrotic range proteinuria of 1661.1 mg/mmol (14.7 mg/mg) proteinuria and serum creatinine of 167.96 *μ*mol/L (1.9 mg/dL) two weeks prior to presentation. The lack of temporal association with a secondary cause would argue for idiopathic MCD.

The patient's baseline serum creatinine was 123.76 *μ*mol/L (1.4 mg/dL) and notably the serum creatinine was 167.96 *μ*mol/L (1.9 mg/dL) seven days prior to his ingestion of the sildenafil. This may have been due to a variable change in volume status, or secondary to the underlying MCD which often has modest changes in serum creatinine on presentation.

Our patient was initially treated with 1 g of methylprednisolone for 3 days followed by steroid taper for his MCD. There is no standard treatment protocol for AIN. The treatment course for this disease process is usually shorter. In this case, the corticosteroids were treating an AIN as well as MCD.

## 4. Conclusion

We present a case of AIN suspected due to sildenafil in an 81-year-old Asian male who also had idiopathic MCD. In the United States, generic formulations of sildenafil are currently available for the treatment of pulmonary hypertension but not erectile dysfunction. The true incidence of renal issues with sildenafil is unknown as there is minimal published or postmarketing data. More cases of AIN may be identified as the number of sildenafil prescriptions increases. Physician awareness for this potential drug-disease association is necessary.
